# 1-4-2: Evaluation of applied mechanical power to individual lungs in a simulator-based setting of one ventilator for two patients

**DOI:** 10.1371/journal.pone.0328813

**Published:** 2025-08-07

**Authors:** Lars-Olav Harnisch, Christian Czock, Matthew A. Levin, Johannes Wieditz, Leif Saager, Konrad Meissner, Onnen Moerer

**Affiliations:** 1 Department of Anaesthesiology, University Medical Center, Georg-August-University, Göttingen, Germany; 2 Department of Anesthesiology, Perioperative and Pain Medicine, Icahn School of Medicine at Mount Sinai, New York, New York, United States of America; 3 Department of Medical Statistics, University Medical Center, Georg-August-University Göttingen, Göttingen, Germany; 4 Outcomes Research Consortium, Cleveland, Ohio, United States of America; Sapienza University of Rome: Universita degli Studi di Roma La Sapienza, ITALY

## Abstract

**Background:**

The concept of ventilating multiple patients concurrently using a single ventilator has been proposed as a solution when the demand for ventilators surpasses the available supply. While the practicality of this approach has been established, a thorough evaluation of the risks involved has yet to be comprehensively addressed.

**Methods:**

Two circuits, a simple one (circuit-1) and another with an adjustable resistance valve (circuit-2), were evaluated within an experimental framework utilizing two computer-controlled lung simulators (TestChest and ASL 5000). These simulators were ventilated by an ICU (intensive care unit) ventilator (Servo-u) employing various ventilation modes (volume- and pressure-controlled ventilation). The study was conducted under differing respiratory conditions, characterized by low compliance (20 ml/cmH_2_O) as well as normal-high compliance (100 ml/cmH_2_O), in order to ascertain the applied tidal volume (VT), pressures, and the resultant mechanical power (MP).

**Results:**

Circuit-1: The applied VT, pressures, and MP differed significantly between the two simulators, as well as in relation to ventilation mode, compliance, and respiratory rate (RR) (p < 0.001); the differences were most pronounced in settings with differing compliance levels. Circuit-2: Differences in VT, pressures, and MP were observed between simulators concerning valve settings (p < 0.001). The VT demonstrated a negative correlation, with volumes derived from valve closure spanning from 50 to 100 ml across all settings. In the design of circuit-2, MP exceeded the 12 J/min threshold in both lung simulators at elevated RR and could only be decreased through valve closure followed by a consequential hypoventilation in one simulator.

**Conclusion:**

The simultaneous ventilation of two patients using a single ventilator is technically viable, yet it presents considerable risks. Even with the integration of an adjustable resistance valve to accommodate varying lung complexities, the likelihood of unilateral hypoventilation and elevated mechanical stress remains high.

## Introduction

The allocation of a single ventilator for use by two patients has received significant attention during the peak of the COVID-19 pandemic [1–9]. The concept of functional separation of a single ventilator, initially proposed in the context of disaster preparedness and unilateral lung pathologies decades ago, obviously continues to maintain its relevance [10–14]. These reports have already highlighted intrinsic issues, such as the absence of individualized monitoring and tailored tidal volumes, inspired oxygen fraction, and positive end-expiratory pressure (PEEP) [15–18]. Only two studies report the concept of ventilator sharing in humans to be feasible [3,19]. In contrast, opinion articles on this topic advocate against the use of ventilator sharing [4,7,20–22]. A consensus statement from several professional societies lists additional concerns: alarm monitoring is not feasible, improvement and deterioration of an individual could go unnoticed, leading to dangerous scenarios for all ventilated patients, the ventilator self-test cannot be performed due to the large tube volume, and therefore measurements are unreliable [7]. In summary, the extant literature indicates that while it is technically feasible to use a single ventilator to support more than one patient, significant concerns regarding the implications, utility, and most critically, safety remain.

We postulated that the practice of ventilator sharing presents significant risks to patients, not solely due to the necessity for relaxation and its implications for the weaning process, but more critically concerning the potential for hypo- or hyperventilation of individual lungs and resultant injurious ventilation. Ventilation-induced lung injury (VILI) has been recognized as a serious problem, and a distinct ventilation strategy has evolved to reduce VILI and ensure lung-protective ventilation [23,24]. Since modern ventilators are not designed to ventilate more than one patient, ventilator settings cannot be adjusted as meticulously as necessary to avoid VILI. This is mainly due to the fact that the parameters measured by the ventilator and used to set the ventilation, such as tidal volume, inspiratory pressure, plateau pressure, and driving pressure, cannot be used in a split ventilator setting because the distribution of volume and pressure between patients is unknown [25]. The rate of energy transfer to the lungs during mechanical ventilation is proposed as a superordinate variable for assessing the impact of ventilation variables on respiratory mechanics conditions and mortality. To examine the potential adverse effects of ventilating multiple patients concurrently on a single ventilator, we conducted a review of existing literature on ventilator sharing and computed the mechanical power using the data available. Furthermore, we evaluated both a basic and an adapted circuit within an experimental framework consisting of one ventilator and two lung simulators. The mechanical ventilation parameters were quantified, and the mechanical power exerted on each lung simulator was computed during discrete mechanical breaths.

## Methods

We searched the MEDLINE database for articles on ventilator sharing/splitting using the key words “ventilator sharing” and “ventilator splitting”. In a second step, we calculated mechanical power, where data were available, based on the formula proposed by Gattinoni et al. [26].

We define a mechanical power of more than 12 J/min as a power with a high probability of causing ventilation-induced lung injury, if the lungs are ventilated for a prolonged period of time [27].

**Fig 1:**
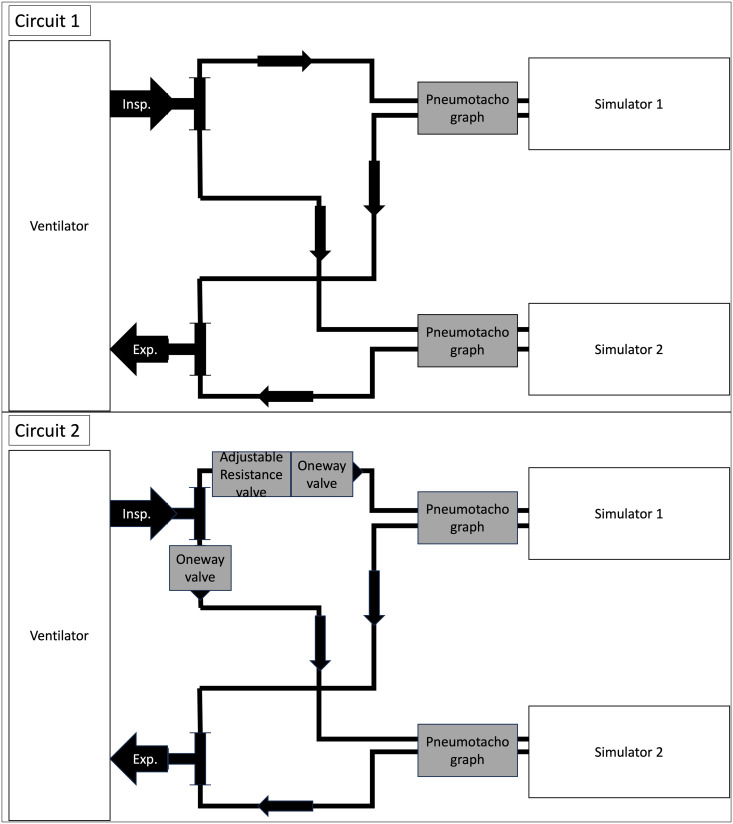
Sketches of the two circuits used.

We used a conventional intensive care unit (ICU) ventilator (Servo-u, Getinge, Solna, Sweden) and two lung simulators (ASL 5000®, IngmarMedical, Pittsburgh, USA and TestChest, Aqai GmbH, Mainz, Germany); two different simulators were used due to availability. Each lung simulator was connected to the ventilator by an individual tubing circuit (model 2158000, Intersurgical, Sank Augustin, Germany; length 3m, diameter 22 mm); in a first-step circuit-1 for both simulators, as a second-step circuit-2 for both simulators. The first circuit was a simple connection of the two lung simulators to the ventilator using two conventional tubes as described by Tonetti et al. [6]. The second circuit consisted of one-way valves and one adjustable resistance valve (custom-designed and -made, Stryker Corporation, USA) to direct and adjust flow within the circuit as proposed by Levin et al. [3] (Fig 1). The work was carried out solely in the laboratory of the Department of Anaesthesiology in Göttingen.

The lung simulators were configured to operate in a passive mode; compliance was set at a low value of 20 ml/cmH_2_O or a normal-high value of 100 ml/cmH_2_O to simulate two degrees of severity, but also to create a clear distinction in this experimental setting; Airway resistance was established at 30 cmH_2_O/l. The ventilation settings were 450 ml of tidal volume per lung in volume-controlled mode, in pressure-controlled mode, the inspiratory pressure was adjusted to achieve 450 ml of tidal volume per lung in circuit 1 at normal-high lung compliance, resulting in an inspiratory pressure of 10 cmH_2_O in our setting, PEEP 8 cmH_2_O, I: E ratio 1: 5, the maximum inspiratory pressure was established at 35 cmH_2_O.

**Fig 2:**
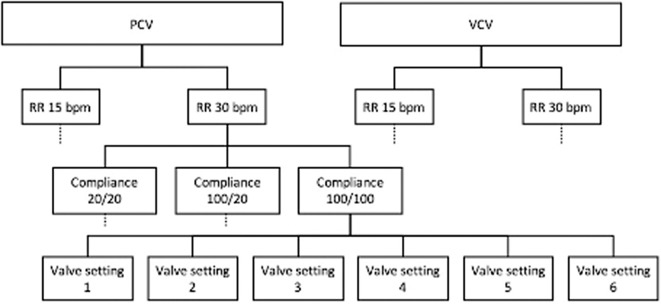
Study protocol. Abbreviations: PCV = pressure controlled ventilation, VCV = volume controlled ventilation, RR = respiratory rate.

Both circuits were tested at both ventilation modes (testing in volume-controlled ventilation (VCV) was for investigative purposes only, valves of circuit-2 are only approved for use in pressure-controlled ventilation (PCV) [28]) and at two different respiratory rates of 15 bpm and 30 bpm, respectively. During the experiment, compliances were first set to identical normal-high values (100 ml/cmH_2_O for both lung simulators), changed to different compliances for both (one lung simulator 20 ml/cmH_2_O, the other 100 ml/cmH_2_O), and finally changed to identical low values (20 ml/cmH_2_O for both lung simulators). For the second circuit, we additionally changed the pressure levels of the adjustable resistance valve to a full scale of possibilities (settings 1–6) in each experimental setting. Resistance changes are caused by the valve by setting six different sized boreholes, which can be selected by turning a screw on the valve. The complete experimental protocol is shown in Fig 2. Signals were recorded with pneumotachographs with differential pressure measurement (Pneumotachograph, 6 Volt, A. Fleisch, Switzerland). Measurement took place within the circuit (in-line) as close to the simulators as possible, i.e., between the tube and the simulator. Each experimental setting was recorded with custom programmed software for 6 minutes at a sampling rate of 100 Hz, of which the first minute of each setting was discarded as a calibration phase and 5 minutes were finally digitally stored. For further handling, the data was saved in a spreadsheet format (Excel, Microsoft, Redmond WA, USA). From the data, we extracted the maximum pressure in the ventilator and each individual lung simulator, we calculated the area under the volume curve in the ventilator and each individual lung simulator as the applied tidal volume, mean airway pressure, and the mechanical power from the equation proposed by Gattinoni et al. [26] applied by each mechanical breath; all values were averaged over all breaths. Furthermore, we conducted evaluations of flow and pressure metrics for each valve configuration to delineate the characteristics of the adjustable resistance valve.

Statistical analysis was performed using SPSS® 27.0 (International Business Machines Corporated [IBM], Armonk, New York, USA) and R version 4.3.1 (R Foundation for Statistical Computing, Vienna, Austria), respectively. Results are presented as mean ± SD, statistical significance was assumed at p < 0.05. Note that, due to the deterministic structure of the experiment, the measurements exhibited a vanishingly small variance. As a result, the assumption of normality is not appropriate.

The paired differences in tidal volume, peak pressure, mean pressure, and mechanical power between the two ventilators have been analyzed using linear models that include time effects to account for repeated measurements.

## Results

### Analysis of published studies

The literature search yielded a total of 46 articles, of which two were duplicates, resulting in 44 articles available for subsequent analysis. All trials defined feasibility as main endpoint, which was confirmed in all trials; none of these trials evaluated the safety or benefit of a shared respiratory circuit. Subsequent calculation of the applied mechanical power was possible in 10 of these 44 studies (see S1 Table). Among the 10 articles, seven present simulator data, one article provides animal data, and two articles encompass human data. There was substantial variation in ventilator settings, including ventilation mode, PEEP, respiratory rate, I:E ratio, across the studies.

The applied mechanical power was injurious in all human and animal trials and all respective settings reported (human: 34.98 ± 12.71 J/min; animal: 24.29 ± 4.22 J/min).

In the simulator trials, the mechanical power was consistently observed to be two or more times lower than that recorded in trials involving patients or animals. (8.78 ± 4.80 J/min; p < 0.001) [2,3,5,6,8,12,15,17,19,29].

### Experimental data

#### Circuit 1.

The applied tidal volumes differed significant between the two simulators, as well as with respect to ventilation mode, compliances, and respiratory rate (all p < 0.001). Tidal volumes differed most in settings of different compliances in both simulators regardless of ventilation mode or respiratory rate. The mean and peak pressures were also significantly different between the simulators in all settings tested (p < 0.001), with one exception (peak pressure: VCV RR 15 Compliance 20/20 differed by 0.014 cmH_2_O (95%-CI: [−0.007, 0.035], p = 0.181). Peak pressures were found to be higher in the VCV mode compared to the PCV mode and at a respiratory rate of 30/min compared to a respiratory rate of 15/min. Applied pressures from the ventilator were at a maximum of Pinsp = 11.78 cmH_2_O/ 13.66 cmH_2_O (PCV/VCV) (Pmean = 6.70 cmH_2_O/ 6.87 cmH_2_O), translating into a maximum pressure in the lung-simulators in the setting of good compliance of 14.12 cmH_2_O, 17.40 cmH_2_O, respectively (PCV/VCV) and a mean pressure in the simulators of 7.89 cmH_2_O, 8.10 cmH_2_O, respectively (PCV/VCV) under the same conditions. Mechanical power differed between simulators in all settings (p < 0.001).

In the context of the study, mechanical power surpassed 12 J/min under the following conditions: ventilation of simulated lungs with reduced compliance at a low respiratory rate, and ventilation of simulated lungs at different levels of compliance across both ventilation modes, including those at a high respiratory rate setting. Details are listed in Table 1.

**Table 1:** Volumes and peak pressures measured in the two simulators in the respective simulated respiratory condition in circuit 1.

	PCV RR 15 Compliance 100/100	PCV RR 15 Compliance 100/20	PCV RR 15 Compliance 20/20	VCV RR 15 Compliance 100/100	VCV RR 15 Compliance 100/20	VCV RR 15 Compliance 20/20	PCV RR 30 Compliance 100/100	PCV RR 30 Compliance 100/20	PCV RR 30 Compliance 20/20	VCV RR 30 Compliance 100/100	VCV RR 30 Compliance 100/20	VCV RR 30 Compliance 20/20
Volume (ml)												
Sim1	330.43 ± 0.72*	188.96 ± 1.11*	355.92 ± 0.997*	326.32 ± 0.72*	231.69 ± 0.51*	343.06 ± 0.69*	330.81 ± 0.97*	272.34 ± 0.58*	332.87 ± 0.60*	319.69 ± 0.89*	283.62 ± 0.67*	337.73 ± 0.86*
Sim2	374.19 ± 0.79*	501.17 ± 0.85*	295.57 ± 0.72*	367.24 ± 0.87*	498.35 ± 1.26*	300.47 ± 0.77*	381.35 ± 1.17*	398.62 ± 0.99*	307.12 ± 0.67*	376.83 ± 1.20*	425.07 ± 1.11*	324.97 ± 0.92*
Peak pressure (cmH_2_O)												
Sim1	8.72 ± 0.02*	10.06 ± 0.05*	15.66 ± 0*	9.35 ± 0.03	10.71 ± 0.02*	16.49 ± 0.02	11.91 ± 0.05*	12.30 ± 0.02*	16.50 ± 0.02*	12.82 ± 0.02*	13.60 ± 0.05*	18.80 ± 0.06*
Sim2	7.61 ± 0.04*	9.39 ± 0.03*	14.81 ± 0.05*	9.35 ± 0.03	11.92 ± 0.05*	16.50 ± 0.05	14.10 ± 0.04*	14.22 ± 0*	16.27 ± 0.01*	17.27 ± 0.05*	18.54 ± 0.06*	21.89 ± 0.06*
Mechanical power (J/min)												
Sim1	6.89 ± 0.02*	3.20 ± 0.05*	7.67 ± 0.03*	6.77 ± 0.02*	4.20 ± 0.01*	7.27 ± 0.02	19.81 ± 0.09*	14.56 ± 0.05*	20.02 ± 0.06*	18.66 ± 0.08*	15.52 ± 0.06*	20.49 ± 0.08*
Sim2	8.25 ± 0.03*	12.80 ± 0.03*	5.88 ± 0.02*	8.03 ± 0.03*	12.69 ± 0.05*	6.02 ± 0.02	24.96 ± 0.13*	26.84 ± 0.60*	17.60 ± 0.06*	24.47 ± 0.13*	29.86 ± 0.13*	19.26 ± 0.09*

All values are mean ± standard deviation. Statistical significance between the simulators within a simulated setting marked ‘*’. Abbreviations: PCV = pressure-controlled ventilation, VCV = volume-controlled ventilation, RR = respiratory rate, Sim = Simulator.

Characterization of the adjustable resistance valve: The valve orifices measured 1 cm each in diameter, with an interstitial spacing of 0.2 cm. Significant variations in flow and pressure were observed across different valve configurations in nearly all experimental scenarios. Due to the ventilator’s maximum pressure threshold of 40 mbar, it was not feasible to assess higher valve settings when coupled with high flow rates of 50 and 60 liters per minute, as detailed in S2 Table.

#### Circuit 2.

**Fig 3:**
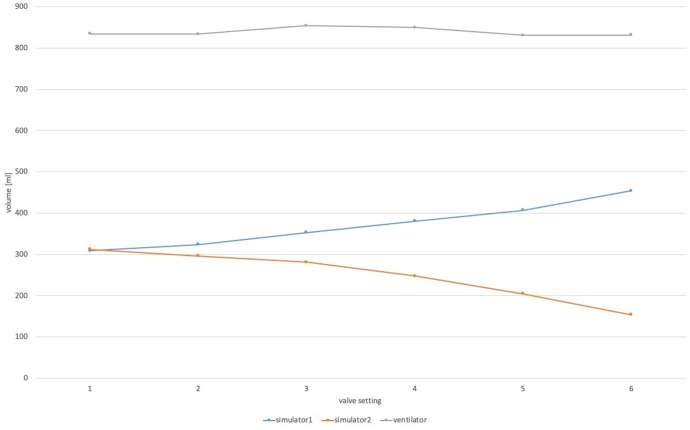
Exemplary volume distribution during the test phase while the valve was closed. While the applied volume by the ventilator remains constant, closing the valve in front of simulator 2 diverts volume towards simulator 1. This pattern was basically found in all settings tested.

The differences in tidal volume were significant (p < 0.001) across the two simulators with the corresponding compliance and respirator settings in relation to valve settings. While adjusting the valve (i.e., turning the valve from setting 1 to setting 6) the resulting volume decreased in simulator 2 while at the same time the resulting volume in simulator 1 increased (Fig 3); this finding was consistent in all settings tested. The aggregate volume administered by the ventilator to the circuit remained unchanged across different valve settings, however, there was a negative correlation observed in the measured volumes.

Significant variation in peak pressures was observed between the simulators concerning valve settings, as well as in tidal volumes (p < 0.001). With closed valve, the peak pressure in one lung simulator decreased while at the same time in the other lung simulator it increased (p < 0.001 for all settings). The peak pressure was higher at higher respiratory rates compared to lower respiratory rates and in VCV as compared to PCV (p < 0.001). The applied pressures of the ventilator were at a maximum of Pinsp = 19.40 cmH_2_O/ 25.66 cmH_2_O (PCV/VCV), translating into peak pressures in the simulators in a fully open valve (valve setting 1) and good compliance of 15.24 cmH_2_O, 17.96 cmH_2_O, respectively (PCV/VCV). The mean pressure in the simulators under the same conditions was 6.49 cmH_2_O and 6.63 cmH_2_O, respectively (PCV/VCV).

As the valve was progressively closed, the resultant mechanical power exhibited an increase in one lung simulator, while concurrently decreasing in the other (p < 0.001). The difference in mechanical power increased significantly (p < 0.001) during valve closure in all combinations of respiratory rate and compliance, except for PCV RR 30/min compliance 20/20 (p = 0.138). Mechanical power remained below 12 J/min in all low respiratory rate settings. Under conditions of elevated respiratory rate ventilation, the mechanical power surpassed the allowable threshold in both lungs. However, beginning at valve setting 5, the MP diminished to below 12 J/min in simulator 2, accompanied by a concurrent decrease in tidal volumes. Detailed results are presented in Table 2.

**Table 2:** Volumes and peak pressures measured and mechanical power calculated at the respective valve settings at the ventilator and the two lung simulators in circuit 2.

		PCV RR 15 Compliance 100/100	PCV RR 15 Compliance 100/20	PCV RR 15 Compliance 20/20	PCV RR 30 Compliance 100/100	PCV RR 30 Compliance 100/20	PCV RR 30 Compliance 20/20	VCV RR 15 Compliance 100/100	VCV RR 15 Compliance 100/20	VCV RR 15 Compliance 20/20	VCV RR 30 Compliance 100/100	VCV RR 30 Compliance 100/20	VCV RR 30 Compliance 20/20
Volume (ml)													
Valve 1#	Sim1	336.09 ± 0.70*	253.55 ± 0.77*	339.86 ± 0.62*	331.21 ± 0.97*	324.49 ± 1.23*	337.04 ± 0.92*	345.54 ± 0.69*	279.81 ± 0.70*	324.73 ± 0.59*	339.97 ± 1.14*	324.49 ± 1.23*	331.49 ± 0.93*
	Sim2	338.13 ± 0.84*	431.14 ± 1.03*	287.35 ± 0.54*	308.02 ± 1.05*	312.18 ± 0.74*	290.15 ± 0.79*	320.31 ± 0.72*	356.56 ± 0.76*	289.13 ± 0.64*	284.54 ± 1.29*	286.78 ± 0.78*	264.21 ± 0.70*
Valve 2^#^	Sim1	372.90 ± 0.64*	253.51 ± 0.75*	340.65 ± 0.59*	359.18 ± 0.81*	337.80 ± 0.97*	337.06 ± 0.91*	360.97 ± 0.75*	283.73 ± 0.72*	335.99 ± 0.68*	342.47 ± 1.18*	337.80 ± 0.97*	333.10 ± 0.77*
	Sim2	341.95 ± 0.62*	429.48 ± 0.72*	286.07 ± 0.55*	300.98 ± 0.82*	295.70 ± 0.92*	290.25 ± 0.75*	302.07 ± 0.99*	351.59 ± 0.89*	276.26 ± 0.54*	282.27 ± 1.25*	272.36 ± 0.95*	262.08 ± 0.84*
Valve 3^#^	Sim1	377.34 ± 0.59*	254.51 ± 0.72*	367.47 ± 0.65*	375.53 ± 1.13*	358.87 ± 0.79*	339.11 ± 0.92*	385.70 ± 0.82*	295.98 ± 0.78*	354.61 ± 0.79*	350.48 ± 1.29*	358.87 ± 0.79*	339.24 ± 0.82*
	Sim2	300.33 ± 0.60*	397.31 ± 0.79*	290.06 ± 0.65*	275.59 ± 0.93*	281.25 ± 0.85*	271.41 ± 0.79*	276.12 ± 0.85*	339.43 ± 0.74*	254.54 ± 0.62*	273.31 ± 1.11*	248.34 ± 0.55*	256.23 ± 0.61*
Valve 4^#^	Sim1	414.17 ± 0.75*	274.57 ± 0.87*	366.43 ± 0.71*	404.81 ± 1.13*	388.72 ± 1.14*	366.23 ± 0.94*	414.59 ± 0.85*	318.57 ± 0.66*	377.44 ± 0.89*	374.22 ± 1.18*	388.27 ± 1.14*	357.52 ± 1.06*
	Sim2	280.38 ± 0.74*	375.70 ± 0.78*	268.76 ± 0.67*	244.58 ± 0.84*	247.35 ± 0.67*	248.36 ± 0.76*	245.10 ± 0.65*	315.05 ± 0.71*	230.45 ± 0.44*	248.74 ± 0.97*	213.41 ± 0.88*	236.20 ± 0.71*
Valve 5^#^	Sim1	412.24 ± 0.78*	300.06 ± 0.77*	393.04 ± 0.63*	446.75 ± 1.47*	384.49 ± 1.14*	392.56 ± 0.82*	408.47 ± 0.77*	340.85 ± 0.88*	411.30 ± 0.85*	393.72 ± 1.72*	384.49 ± 1.14*	383.53 ± 1.03*
	Sim2	298.20 ± 0.81*	359.20 ± 0.78*	248.87 ± 0.78*	202.47 ± 1.04*	204.80 ± 0.71*	215.46 ± 0.62*	250.97 ± 0.85*	291.89 ± 0.65*	195.51 ± 0.64*	227.97 ± 1.24*	217.58 ± 0.86*	209.25 ± 0.88*
Valve 6^#^	Sim1	453.43 ± 0.77*	351.70 ± 0.74*	417.76 ± 0.92*	461.94 ± 1.43*	415.34 ± 1.05*	428.75 ± 0.93*	455.94 ± 0.67*	401.07 ± 0.75*	429.13 ± 0.84*	438.53 ± 1.25*	415.34 ± 1.05*	420.17 ± 0.94*
	Sim2	238.00 ± 0.61*	294.12 ± 0.52*	229.65 ± 0.87*	165.31 ± 1.13*	152.79 ± 1.08*	183.05 ± 0.71*	203.23 ± 0.78*	225.63 ± 0.59*	175.24 ± 0.67*	182.95 ± 0.90*	181.05 ± 0.61*	169.13 ± 0.80*
Pressure (cmH_2_O)													
Valve 1^#^	Sim1	9.79 ± 0.05*	13.13 ± 0.00*	15.65 ± 0.01*	15.10 ± 0.06*	17.62 ± 0.05*	18.28 ± 0.05*	10.83 ± 0.03*	14.82 ± 0.03*	16.62 ± 0.05*	17.80 ± 0.06*	20.15 ± 0.08*	20.96 ± 0.07*
	Sim2	7.84 ± 0.05*	9.05 ± 0.05*	14.08 ± 0.05*	11.34 ± 0.03*	11.41 ± 0.05*	16.58 ± 0.02*	8.30 ± 0.05*	9.0 ± 0.02*	15.00 ± 0.05*	11.76 ± 0.02*	11.88 ± 0.04*	16.66 ± 0.04*
Valve 2^#^	Sim1	10.63 ± 0.04*	13.13 ± 0.01*	15.66 ± 0.01*	16.06 ± 0.04*	18.18 ± 0.02*	18.26 ± 0.05*	11.15 ± 0.04*	14.92 ± 0.04*	17.00 ± 0.04*	17.91 + 0.07 *	20.99 ± 0.08*	21.03 ± 0.08*
	Sim2	7.98 ± 0.03*	9.01 ± 0.03*	13.98 ± 0.05*	11.24 ± 0.03*	10.95 ± 0.03*	16.58 ± 0.02*	7.99 ± 0.04*	8.91 ± 0.04*	14.45 ± 0.05*	11.66 ± 0.03*	11.54 ± 0.04*	16.57 ± 0.03*
Valve 3^#^	Sim1	10.67 ± 0.05*	13.13 ± 0.00*	16.19 ± 0.05*	16.93 ± 0.07*	19.25 ± 0.05*	18.26 ± 0.05*	11.87 ± 0.03*	15.34 ± 0.04*	17.66 ± 0.06*	18.34 ± 0.07*	22.46 ± 0.08*	21.36 ± 0.08*
	Sim2	7.44 ± 0.04*	8.60 ± 0.03*	14.17 ± 0.05*	10.55 ± 0.05*	10.63 ± 0.02*	15.73 ± 0.05*	7.61 ± 0.05*	8.78 ± 0.04*	13.56 ± 0.06*	11.42 ± 0.05*	10.84 ± 0.02*	16.19 ± 0.05*
Valve 4^#^	Sim1	10.95 ± 0.05*	13.87 ± 0.02*	16.15 ± 0.06*	17.74 ± 0.04*	20.91 ± 0.07*	19.64 ± 0.06*	12.31 ± 0.04*	16.16 ± 0.05*	18.19 ± 0.04*	19.64 ± 0.06*	24.62 ± 0.09*	22.51 ± 0.08*
	Sim2	7.15 ± 0.02*	8.34 ± 0.04*	13.30 ± 0.01*	9.71 ± 0.02*	9.77 ± 0.05*	14.67 ± 0.05*	7.15 ± 0.00*	8.40 ± 0.04*	12.48 ± 0.02*	10.73 ± 0.02*	9.76 ± 0.05*	15.19 ± 0.05*
Valve 5^#^	Sim1	10.95 ± 0.05*	14.60 ± 0.01*	16.89 ± 0.04*	19.50 ± 0.06*	22.55 ± 0.06*	21.27 ± 0.06*	12.22 ± 0.05*	16.96 ± 0.05*	19.35 ± 0.07*	20.91 ± 0.07*	24.33 ± 0.10*	24.32 ± 0.09*
	Sim2	7.44 ± 0.04*	8.09 ± 0.04*	12.55 ± 0.05*	8.70 ± 0.03*	8.65 ± 0.05*	13.25 ± 0.05*	7.25 ± 0.02*	7.97 ± 0.00*	10.85 ± 0.03*	10.08 ± 0.05*	9.85 ± 0.05*	13.81 ± 0.02*
Valve 6^#^	Sim1	11.58 ± 0.04*	16.06 ± 0.04*	17.63 ± 0.06*	20.34 ± 0.07*	25.62 ± 0.06*	23.57 ± 0.05*	13.04 ± 0.04*	18.71 ± 0.03*	20.07 ± 0.07*	23.52 ± 0.10*	26.92 ± 0.11*	27.21 ± 0.10*
	Sim2	6.54 ± 0.02*	7.24 ± 0.03*	11.76 ± 0.01*	7.74 ± 0.04*	7.36 ± 0.02*	11.75 ± 0.03*	6.42 ± 0.04*	6.83 ± 0.04*	10.02 ± 0.00*	8.80 ± 0.03*	8.79 ± 0.01*	11.70 ± 0.05*
Mechanical power (J/min)													
Valve 1^#^	Sim1	7.06 ± 0.02*	4.75 ± 0.02*	7.18 ± 0.02*	19.85 ± 0.09*	17.72 ± 0.06*	20.42 ± 0.09*	7.34 ± 0.02*	5.45 ± 0.02*	6.72 ± 0.02*	20.71 ± 0.11*	19.22 ± 0.12*	19.88 ± 0.09*
	Sim2	7.12 ± 0.03*	10.18 ± 0.04*	5.65 ± 0.02*	17.68 ± 0.10*	18.05 ± 0.07*	16.08 ± 0.07*	6.59 ± 0.02*	7.69 ± 0.03*	5.70 ± 0.02*	15.59 ± 0.11*	15.78 ± 0.07*	13.90 ± 0.06*
Valve 2^#^	Sim1	8.21 ± 0.02*	4.75 ± 0.02*	7.20 ± 0.02*	22.63 0.08*	19.12 ± 0.06*	20.42 ± 0.09*	7.83 ± 0.02*	5.55 ± 0.02*	7.06 ± 0.02*	20.95 ± 0.12*	20.50 ± 0.09*	20.04 ± 0.07*
	Sim2	7.24 ± 0.02*	10.12 ± 0.03*	5.62 ± 0.02*	17.04 ± 0.07*	16.57 ± 0.06*	16.09 ± 0.07*	6.06 ± 0.03*	7.53 ± 0.03*	5.35 ± 0.02*	15.40 ± 0.11*	14.56 ± 0.08*	13.72 ± 0.07*
Valve 3^#^	Sim1	8.35 ± 0.02*	4.78 ± 0.02*	8.04 ± 0.02*	24.34 ± 0.12*	21.97 ± 0.08*	20.62 ± 0.09*	8.63 ± 0.03*	5.89 ± 0.02*	7.63 ± 0.03*	21.75 ± 0.13*	22.61 ± 0.08*	20.64 ± 0.08*
	Sim2	6.01 ± 0.02*	9.01 ± 0.03*	5.73 ± 0.02*	14.83 ± 0.08*	15.31 ± 0.07*	14.49 ± 0.06*	5.34 ± 0.02*	7.16 ± 0.02*	4.78 ± 0.02*	14.64 ± 0.09*	12.62 ± 0.04*	13.25 ± 0.05*
Valve 4^#^	Sim1	9.59 ± 0.03*	5.30 ± 0.02*	8.00 ± 0.02*	27.54 ± 0.13*	24.81 ± 0.07*	23.37 ± 0.10*	9.60 ± 0.03*	6.54 ± 0.02*	8.36 ± 0.03*	24.20 ± 0.12*	25.72 ± 0.12*	22.47 ± 0.11*
	Sim2	5.46 ± 0.02*	8.29 ± 0.02*	5.15 ± 0.02*	12.33 ± 0.07*	12.54 ± 0.05*	12.63 ± 0.06*	4.53 ± 0.02*	6.43 ± 0.02*	4.17 ± 0.01*	12.65 ± 0.08*	10.03 ± 0.06*	11.70 ± 0.05*
Valve 5^#^	Sim1	9.51 ± 0.03*	6.01 ± 0.02*	8.87 ± 0.02*	32.45 ± 0.18*	27.77 ± 0.09*	26.18 ± 0.09*	9.39 ± 0.03*	7.21 ± 0.03*	9.49 ± 0.03*	26.30 ± 0.19*	25.30 ± 0.12*	25.20 ± 0.11*
	Sim2	5.95 ± 0.02*	7.77 ± 0.02*	4.63 ± 0.02*	9.27 ± 0.07*	9.43 ± 0.04*	10.17 ± 0.04*	4.68 ± 0.02*	5.77 ± 0.02*	3.35 ± 0.01*	11.08 ± 0.09*	10.32 ± 0.06*	9.74 ± 0.06*
Valve 6^#^	Sim1	10.98 ± 0.03*	7.54 ± 0.02*	9.71 ± 0.03*	34.32 ± 0.18*	33.30 ± 0.12*	30.30 ± 0.11*	11.08 ± 0.02*	9.14 ± 0.03*	10.11 ± 0.03*	15.98* ± 0.06	28.74 ± 0.12*	29.30 ± 0.11*
	Sim2	4.36 ± 0.02*	5.84 ± 0.02*	4.15 ± 0.02*	6.89 ± 0.07*	6.16 ± 0.06*	7.99 ± 0.05*	3.52 ± 0.02*	4.05 ± 0.01*	2.91 ± 0.01*	5.29 ± 0.03*	7.86 ± 0.04*	7.13 ± 0.05*

All values are mean ± standard deviation, statistical significance (p < 0.001) between simulators within one simulated setting marked “*”.

## Discussion

In times of actual or imminent shortages of ventilator availability, the concept of sharing a ventilator between a variable number of patients gained renewed interest [3,5,6,8,9,20,30,31]. While this solution appears practical from the standpoint of disaster management, it constitutes a hazardous approach when considering the pulmonary mechanics and pathophysiology associated with acute respiratory failure.

For clinical application, multiple significant considerations must be accounted for. Primarily, paralysis is indispensable in all patients to ensure uninterrupted mandatory ventilation, negating spontaneous respiratory efforts. Optimization of lung size matching is critical since the ventilatory volume accessible is specific to the individual and may vary dynamically, particularly with the “baby lung” concept [32]. When utilizing a single ventilator, the lung demonstrating superior compliance is prone to hyperventilation, whereas the lung with inferior compliance may experience hypoventilation, thereby posing a risk to both patients.

In our experimental setup, we first measured volumes and pressures and calculated mechanical power in a very basic circuit without the possibility of adjusting applied mechanical ventilation between patients [6]. The tidal volumes exhibited significant variation between the two lung simulators when compliance varied between the lungs, particularly at lower respiratory rates. It was observed that the volumes measured in the more compliant lung were double those in the less compliant lung. This outcome is unsurprising, given the increased time available to deliver volume.

This finding confirms our hypothesis that a less compliant lung will be hypoventilated. Interestingly, our data did not confirm the overinflation of the more compliant lung, which could be due to the fact that we measured in simulators rather than in actual lungs. A clinical trial that measures two patients with marked differences in lung compliance ventilated with one ventilator could confirm our findings, but it is unlikely to be carried out due to serious ethical concerns. When compliance was not matched, applied volumes and pressures differed significantly between both lung-simulators (Table 1). Mechanical power remained safe in low respiratory rates, but increased significantly in high respiratory rate scenarios, potentially exposing lungs to severe lung injury.

To account for differences in compliance between the two simulated lungs ventilated by the same ventilator, different techniques have been proposed [3,5,8–10,14,20,29–31,33–35]. The limitations of all these alternatives lie in their relatively static or non-dynamic characteristics. Since compliance varies rapidly in acute respiratory distress syndrome (ARDS), regular and meticulous adjustments must be made to ensure adequate ventilation for both patients. To account for the different mechanics of the lung that could change rapidly, Levin et al. [3] proposed adjustable resistance valves that can be inserted into the circuit and, by closing the valve, direct the flow between the two lungs. The advantages of these valves include the option to 3D print them, making them readily available, light, small, robust, easy to use, and operated without electrical power. All these characteristics make them more attractive than a stainless steel threaded sleeve diaphragm valve or a Hoffman clamp, which has also been proposed [5,33]. Additionally, the valve settings are adjustable, the valves are disposable or can be sterilized, and the material is potentially recyclable. All of these benefits, including the expected straightforward costs, also make these valves interesting for countries with already limited healthcare resources that might consider sharing ventilators much earlier during a crisis. Levin et al. found that the applied volumes can be directed between the two ventilated subjects using this adjustable resistance valve [3].

Our results confirm their general findings that applied volumes can be directed in favor of one subject. In our experimental setting with a low respiratory rate, approximately 100 ml of volume could be diverted by closing the valve to setting 6 (Table 2). The resulting changes in peak pressure and mechanical power between ventilated simulators with a low respiratory rate while the valve was closed were statistically significant but clinically negligible. Our results confirm feasibility; however, the clinical relevance of diversion of such small volumes is questionable. Moreover, in instances of elevated respiratory rates, the volume diversion was limited to approximately 40 ml. This limited volume diversion comes with the price of inducing ventilation-induced lung injury. Mechanical power was increased above 12 J/min in all settings with a high respiratory rate of 30 bpm, in some scenarios almost three times. This suggests that in clinical reality, not only one but actually two patients are exposed to the risk of VILI at the same time. Mechanical power was finally reduced below the assumed safe threshold in valve settings 5 and 6 especially at high respiratory rates at the cost of dangerous hypoventilation (tidal volumes of roughly 150 ml with an intended 450 ml). To detect this in a clinical setting, first, the measurement of flow and pressures must take place close to the patient rather than close to/in the ventilator, and second, mechanical power must be calculated and easily displayed to caregivers. Taking into account this clinical reality, our data raise serious concerns about the safety of two patients when considering ventilator sharing.

Previous trials evaluating ventilator sharing only confirmed feasibility; none of these trials explicitly evaluated safety. We retrospectively calculated the mechanical power of these trials, where possible (S1 Table). An injurious level of mechanical power was observed in all documented trials involving both human subjects and animals. Simulator trials reported mechanical power almost universally two to three times lower compared to trials in patients or animals [2,3,5,6,8,12,15,19,29]. This is probably due to methodological reasons. We cautiously conclude that mechanical power might be significantly underestimated in some of these studies. Trials that used study designs with ventilator settings close to our settings also report higher mechanical power, more in line with our findings.

Our findings, which utilize two advanced lung simulators with finely adjustable compliance alongside a critical care ventilator frequently employed in clinical practice, provide new evidence indicating that the current equipment does not facilitate adequate and, crucially safe ventilation for two patients with a single ventilator [7].

We recommend exercising prudence in the utilization of ventilator-sharing for multiple justifiable reasons. Firstly, it is infeasible to customize ventilation in this context concerning respiratory rate, which consequently precludes the specific adjustment for respiratory acid-base imbalances. Second, it is almost impossible to individualize ventilation with respect to PEEP. Our findings indicate that even with the implementation of an adjustable resistance valve, precise volumetric settings remain unattainable. Furthermore, despite intentions aimed at optimizing outcomes, adjustments made to the valve settings to redirect volume towards the subject with inferior lung compliance result in an escalation of mechanical power, which can potentially precipitate ventilation-induced lung injury. Conversely, the volumes redirected are minimal, resulting in further hypoventilation of the lung characterized by poorer compliance.

In circumstances where the implementation of ventilator-sharing in clinical practice becomes unavoidable, the following considerations must be taken into account. Lung size and compliance should be matched, low respiratory rate and pressure-controlled mode should be used, and flow and pressure should be measured close to patient. If lung compliance becomes increasingly deviant and sufficient diversion of tidal volume is no longer possible, ventilation should be changed to individual ventilators for each subject rather sooner than later. Circuits without the option to make adjustments should not be used.

This is an experimental trial conducted under laboratory conditions and with two computer-operated lung simulators. This setting can only roughly reflect the clinical reality. However, the setup and placement of our sensors a) made us independent of the values measured by the ventilator, the lung simulators, respectively, and b) allowed us to test for extreme compliances and valve settings without risk to patients.

A discrepancy of approximately 200 ml was observed between the volume administered by the ventilator and the cumulative volumes recorded at the simulators. This phenomenon has been previously documented and is likely attributed to the underestimation of the considerable volume of the tubing system by the ventilator.

Within this experimental framework, the minimal dispersion of the measured values highlights the quality and precision of our measurement setup and the sensors employed; however, this may consequently exaggerate disparities and significance.

The threshold we used for injurious mechanical power (<12 J/min) is rather conservative. We used this threshold because ventilation-induced lung sequelae were reported at this value [27,36]. More recent trials report worsening of patient-centered outcomes at more liberal thresholds of 17 J/min [37] and 22 J/min respectively. We use this conservative threshold because we evaluated injurious ventilation, not patient outcome. However, even at a threshold of 17 J/min, our results and conclusions apply.

## Conclusion

The simultaneous ventilation of two patients using a single ventilator is technically achievable. However, the presence of minor, clinically insignificant diverted volumes, significant hypoventilation of one lung, and the possibility of harmful mechanical power output—particularly at elevated respiratory rates—pose risks to patients. Our findings advise against the practice of ventilator sharing among patient populations with pulmonary pathologies.

**QUICK LOOK**

Current knowledge: Simultaneous ventilation of multiple patients with one ventilator has been suggested when the number of ventilators needed exceeds the available resources. Although feasibility has been demonstrated, the associated risks have not been considered comprehensive.

What this paper contributes to our knowledge: In situations of different compliance between ventilated patients with one ventilator, serious hypoventilation of one lung and potentially harmful mechanical power output result; the introduction of an adjustable valve can only divert small, clinically negligible volumes and does not alter injuriousness.

Ventilating two patients with one ventilator is technically feasible, but with substantial risks and is therefore discouraged.

## Supporting information

S1 Table Mechanical Power calculations from the available literature. For all calculations the original formula provided by Gattinoni et al. [26] was used.
(DOCX)

S2 Table Resistance values of the adjustable resistance valve at the different valve settings and at different flowrates. (DOCX)

## Abbreviations

ARDS: acute respiratory distress syndrome
ICU: Intensive care unit
MP: mechanical power
PCV: pressure-controlled ventilation
PEEP: positive end-expiratory pressure
RR: respiratory rate
VCV: volume-controlled ventilation
VILI: ventilation-induced lung injury
VT: tidal volume
