# Supplementary material for: 1-4-2: Evaluation of applied mechanical power to individual lungs in a simulator-based setting of one ventilator for two patients
Source: PLoS One. 2025 Aug 7;20(8):e0328813. doi: 10.1371/journal.pone.0328813 (PMC12331122; doi:10.1371/journal.pone.0328813)
Supplement: S1 Table — For all calculations the original formula provided by Gattinoni et al. [26] was used. (DOCX) [file pone.0328813.s001.docx]

S1 Table: Mechanical Power calculations from the availiable literature. For all calculations the original formula provided by Gattinoni et al [26]. was used.

| Source | **Power humans**  **[J/min]** | **Power simulators**  **[J/min]** | **Power animals**  **[J/min]** |
| --- | --- | --- | --- |
| Beitler et al.^10^ Patient 1A | 20.18 |  |  |
| Beitler et al. Patient 1B | 15.19 |  |  |
| Beitler et al. Patient 2A | 21.17 |  |  |
| Beitler et al. Patient 2B | 37.55 |  |  |
| Beitler et al. Patient 3A | 26.28 |  |  |
| Beitler et al. Patient 3B | 20.31 |  |  |
| Branson et al. ^7^ Simulation 1-1 VCV |  | 4.12 |  |
| Branson et al. Simulation 1-2 VCV |  | 4.08 |  |
| Branson et al. Simulation 1-3 VCV |  | 4.15 |  |
| Branson et al. Simulation 1-4 VCV |  | 4.12 |  |
| Branson et al. Simulation 2-1 VCV |  | 2.45 |  |
| Branson et al. Simulation 2-2 VCV |  | 3.28 |  |
| Branson et al. Simulation 2-3 VCV |  | 4.93 |  |
| Branson et al. Simulation 2-4 VCV |  | 5.82 |  |
| Branson et al. Simulation 3-1 VCV |  | 4.69 |  |
| Branson et al. Simulation 3-2 VCV |  | 4.43 |  |
| Branson et al. Simulation 3-3 VCV |  | 4.43 |  |
| Branson et al. Simulation 3-4 VCV |  | 4.44 |  |
| Branson et al. Simulation 4-1 VCV |  | 4.10 |  |
| Branson et al. Simulation 4-2 VCV |  | 4.66 |  |
| Branson et al. Simulation 4-3 VCV |  | 3.23 |  |
| Branson et al. Simulation 4-4 VCV |  | 5.22 |  |
| Branson et al. Simulation 1-1 VCV |  | 5.60 |  |
| Branson et al. Simulation 1-2 VCV |  | 5.55 |  |
| Branson et al. Simulation 1-3 VCV |  | 5.64 |  |
| Branson et al. Simulation 1-4 VCV |  | 5.63 |  |
| Branson et al. Simulation 2-1 VCV |  | 3.34 |  |
| Branson et al. Simulation 2-2 VCV |  | 4.42 |  |
| Branson et al. Simulation 2-3 VCV |  | 6.63 |  |
| Branson et al. Simulation 2-4 VCV |  | 7.14 |  |
| Branson et al. Simulation 3-1 VCV |  | 5.68 |  |
| Branson et al. Simulation 3-2 VCV |  | 5.40 |  |
| Branson et al. Simulation 3-3 VCV |  | 5.59 |  |
| Branson et al. Simulation 3-4 VCV |  | 5.48 |  |
| Branson et al. Simulation 4-1 VCV |  | 5.58 |  |
| Branson et al. Simulation 4-2 VCV |  | 6.41 |  |
| Branson et al. Simulation 4-3 VCV |  | 4.41 |  |
| Branson et al. Simulation 4-4 VCV |  | 7.40 |  |
| Herrmann et al.^27^ Situation 1 patient 1 |  | 6.88 |  |
| Herrmann et al. Situation 2 patient 1 |  | 3.84 |  |
| Herrmann et al. Situation 3 patient 1 |  | 7.03 |  |
| Herrmann et al. Situation 4 patient 1 |  | 3.19 |  |
| Herrmann et al. Situation 5 patient 1 |  | 12.95 |  |
| Herrmann et al. Situation 6 patient 1 |  | 6.89 |  |
| Herrmann et al. Situation 7 patient 1 |  | 19.65 |  |
| Herrmann et al. Situation 8 patient 1 |  | 10.82 |  |
| Herrmann et al. Situation 9 patient 1 |  | 12.79 |  |
| Herrmann et al. Situation 10 patient 1 |  | 10.93 |  |
| Herrmann et al. Situation 1 patient 2 |  | 6.95 |  |
| Herrmann et al. Situation 2 patient 2 |  | 4.08 |  |
| Herrmann et al. Situation 3 patient 2 |  | 12.83 |  |
| Herrmann et al. Situation 4 patient 2 |  | 7.34 |  |
| Herrmann et al. Situation 5 patient 2 |  | 10.30 |  |
| Herrmann et al. Situation 6 patient 2 |  | 7.18 |  |
| Herrmann et al. Situation 7 patient 2 |  | 6.99 |  |
| Herrmann et al. Situation 8 patient 2 |  | 3.06 |  |
| Herrmann et al. Situation 9 patient 2 |  | 7.34 |  |
| Herrmann et al. Situation 10 patient 2 |  | 4.07 |  |
| Levin et al.^1^ Study 1 patient A time 0 | 32.80 |  |  |
| Levin et al. Study 1 patient A time 30 | 33.81 |  |  |
| Levin et al. Study 1 patient A time 60 | 47.73 |  |  |
| Levin et al. Study 1 patient B time 0 | 56.14 |  |  |
| Levin et al. Study 1 patient B time 30 | 55.89 |  |  |
| Levin et al. Study 1 patient B time 60 | 54.96 |  |  |
| Levin et al. Study 2 patient A time 0 | 40.12 |  |  |
| Levin et al. Study 2 patient A time 30 | 39.71 |  |  |
| Levin et al. Study 2 patient A time 60 | 38.92 |  |  |
| Levin et al. Study 2 patient B time 0 | 25.02 |  |  |
| Levin et al. Study 2 patient B time 30 | 29.34 |  |  |
| Levin et al. Study 2 patient B time 60 | 34.53 |  |  |
| Neyman et al.^6^ PCV |  | 15.25 |  |
| Neyman et al. VCV |  | 18.13 |  |
| Stiers et al.^25^ Animal 1 |  |  | 21.31 |
| Stiers et al. Animal 2 |  |  | 27.28 |
| Tonetti et al.^2^ Situation 1 |  | 14.34 |  |
| Tonetti et al. Situation 2 |  | 16.73 |  |
| Tonetti et al. Situation 3 |  | 10.09 |  |
| Tonetti et al. Situation 4 |  | 14.68 |  |
| Tonetti et al. Situation 5 |  | 15.16 |  |
| Han et al.^28^ Number 1 lung A |  | 4.26 |  |
| Han et al. Number 1 lung B |  | 5.04 |  |
| Han et al. Number 2 lung A |  | 15.67 |  |
| Han et al. Number 2 lung B |  | 17.41 |  |
| Han et al. Number 3 lung A |  | 12.73 |  |
| Han et al. Number 3 lung B |  | 19.18 |  |
| Han et al. Number 4 lung A |  | 4.65 |  |
| Han et al. Number 4 lung B |  | 7.15 |  |
| Otero et al. SIM1, C45 |  | 5.54 |  |
| Otero et al. SIM1, C30 |  | 7.79 |  |
| Otero et al. SIM1, C20 |  | 9.57 |  |
| Otero et al. SIM1, C10 |  | 11.40 |  |
| Otero et al. SIM1, C10 |  | 12.82 |  |
| Otero et al. SIM2, C45 |  | 4.96 |  |
| Otero et al. SIM2, C30 |  | 17.26 |  |
| Otero et al. SIM2, C20 |  | 14.25 |  |
| Otero et al. SIM2, C10 |  | 10.81 |  |
| Otero et al. SIM2, C10 |  | 13.52 |  |
| Colombo et al.^8^ setting C 50-50/ R 5-5 simulator 1 |  | 14.88 |  |
| Colombo et al. setting C 50-50/ R 5-20 simulator 1 |  | 15.41 |  |
| Colombo et al. setting C 50-30/ R 20-20 simulator 1 |  | 16.36 |  |
| Colombo et al. setting C 50-30/ R 5-20 simulator 1 |  | 16.10 |  |
| Colombo et al. setting C 50-30/ R 20-5 simulator 1 |  | 15.21 |  |
| Colombo et al. setting C 50-50/ R 5-5 simulator 2 |  | 15.41 |  |
| Colombo et al. setting C 50-50/ R 5-20 simulator 2 |  | 16.66 |  |
| Colombo et al. setting C 50-30/ R 20-20 simulator 2 |  | 11.46 |  |
| Colombo et al. setting C 50-30/ R 5-20 simulator 2 |  | 10.75 |  |
| Colombo et al. setting C 50-30/ R 20-5 simulator 2 |  | 9.81 |  |
