# Supplementary material for: 1-4-2: Evaluation of applied mechanical power to individual lungs in a simulator-based setting of one ventilator for two patients
Source: PLoS One. 2025 Aug 7;20(8):e0328813. doi: 10.1371/journal.pone.0328813 (PMC12331122; doi:10.1371/journal.pone.0328813)
Supplement: S2 Table — (DOCX) [file pone.0328813.s002.docx]

S2 Table: Resistance values of the adjustable resistance valve at the different valve settings and at different flowrates

| Valve setting | Resistance R [mbar x s/l] | | | | | |
| --- | --- | --- | --- | --- | --- | --- |
|  | 15 liter | 20 liter | 30 liter | 40 liter | 50 liter | 60 liter |
| 1 | n/a | 4 | 7.2 | 9.5 | 11.48 | 15.33 |
| 2 | 1.47 | 6.5 | 10.07 | 11.8 | 16.16 | 22 |
| 3 | 3.87 | 8.1 | 12.13 | 14.6 | 19.84 | 35.22 |
| 4 | 6.8 | 14 | 22.6 | 23.95 | 31.6 | 38.9 |
| 5 | 13.2 | 20.5 | 27.73 | 43.9 | 45.04 | n/a |
| 6 | 43.2 | 48.7 | 64.27 | 62.25 | 11.48 | n/a |
